# Supplementary figures and images for: Inhibition of the H3K27 demethylase UTX enhances the epigenetic silencing of HIV proviruses and induces HIV-1 DNA hypermethylation but fails to permanently block HIV reactivation
Source: PLoS Pathog. 2021 Oct 21;17(10):e1010014. doi: 10.1371/journal.ppat.1010014 (PMC8562785; doi:10.1371/journal.ppat.1010014)

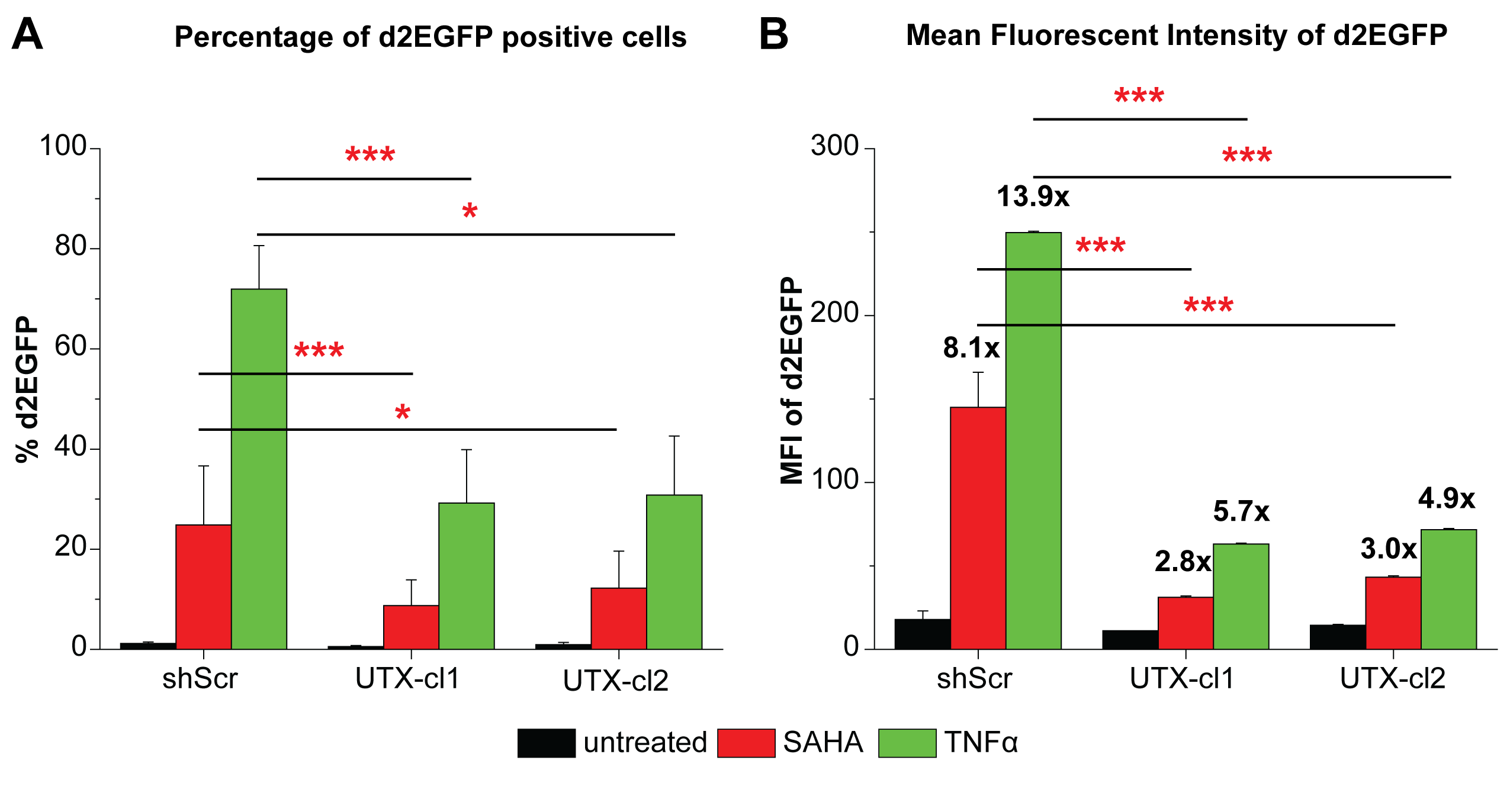

Supplement: S1 Fig — (A) percentage of d2EGFP positive subpopulation of cells versus (B) arithmetic mean of fluorescence intensity of d2EGFP in the population. Fold induction was calculated relative to untreated condition for each shRNA treatment. (TIF) [file ppat.1010014.s001.tif]

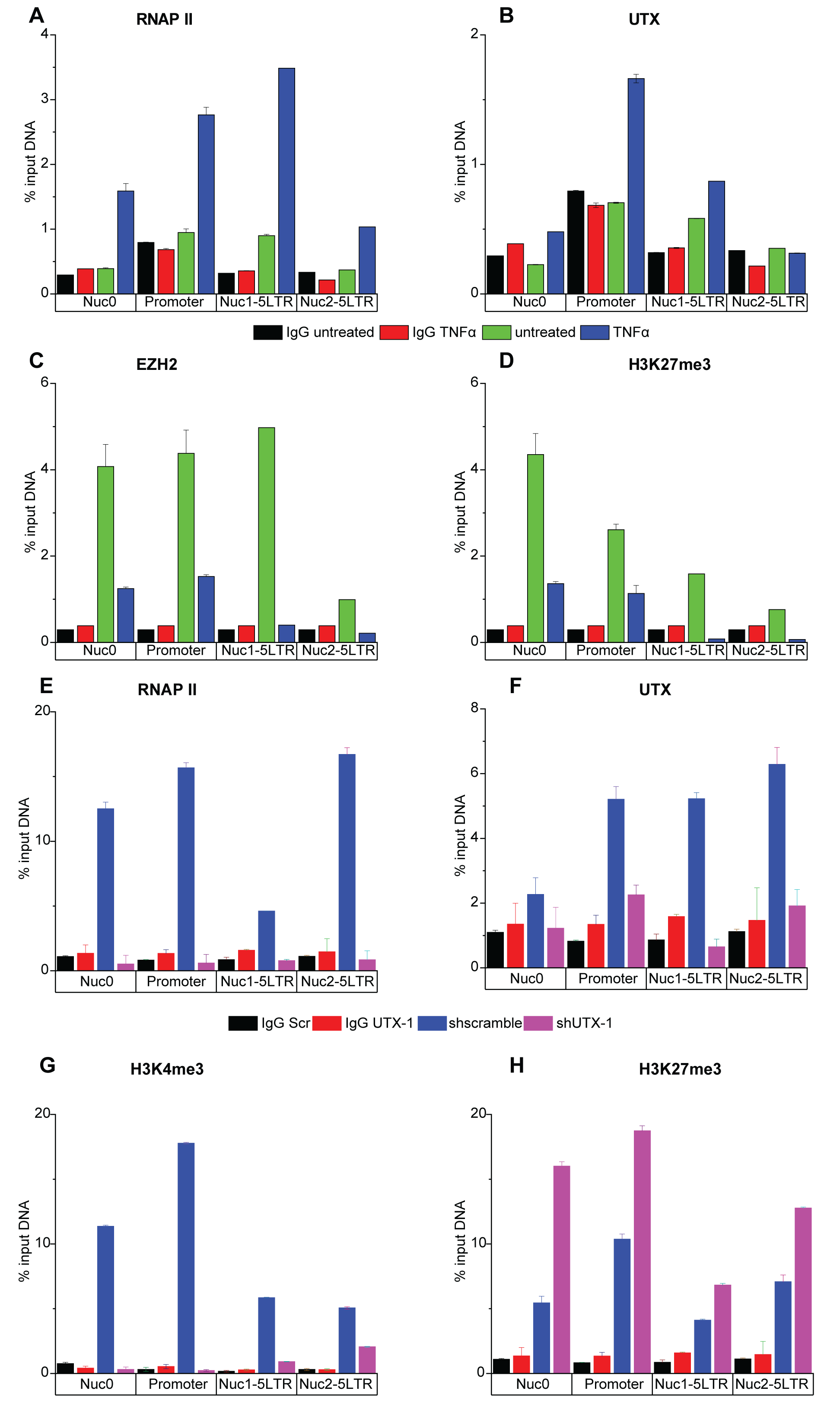

Supplement: S2 Fig — ChIP assays measuring the enrichments of (A) RNAPII, (B) UTX, (C) EZH2, and (D) H3K27me3 at the Nuc-0, HIV-1 promoter, Nuc-1, and Nuc-2 regions of latent or reactivated HIV-1. Latent proviruses in E4 cells were reactivated by TNFα (10 ng/ml) for an hour. Cells were treated with ethylene glycol bis (succinimidyl succinate) (EGS) (1.5 mM) for 30 minutes and subsequently crosslinked with formaldehyde (1%) for an additional 10 minutes. ChIP assays measuring the enrichment of (E) RNAPII, (F) UTX, (G) H3K4me3, and (H) H3K27me3 along HIV-1 genome in one UTX knocked down clone. Error bars: SEM of 3 separate quantitative real-time PCRs. The same amount of rabbit IgG was used for immunoprecipitation per each treatment condition. Primers used for qPCR: HIV-1 Nuc-0: -390F, ACA CAC AAG GCT ACT TCC CTG A, and -283 R, TCT ACC TTA TCT GGC TCA ACT GGT; HIV-1 promoter: -116 F, AGC TTG CTA CAA GGG ACT GGT, and +4 R, ACC CAG TAC AGG CAA AAA GCA G; HIV-1 Nuc-1: +30 F, CTG GGA GCT CTC TGG CTA ACT A, and Nuc1- R, TTT CAA GTC CCT GTT CGG GCG and HIV-1 Nuc-2: +283 F, GAC TGG TGA GTA CGC CAA AAA T, and +390 R, TTT CCC ATC GCG ATC TAA TTC. (TIF) [file ppat.1010014.s002.tif]

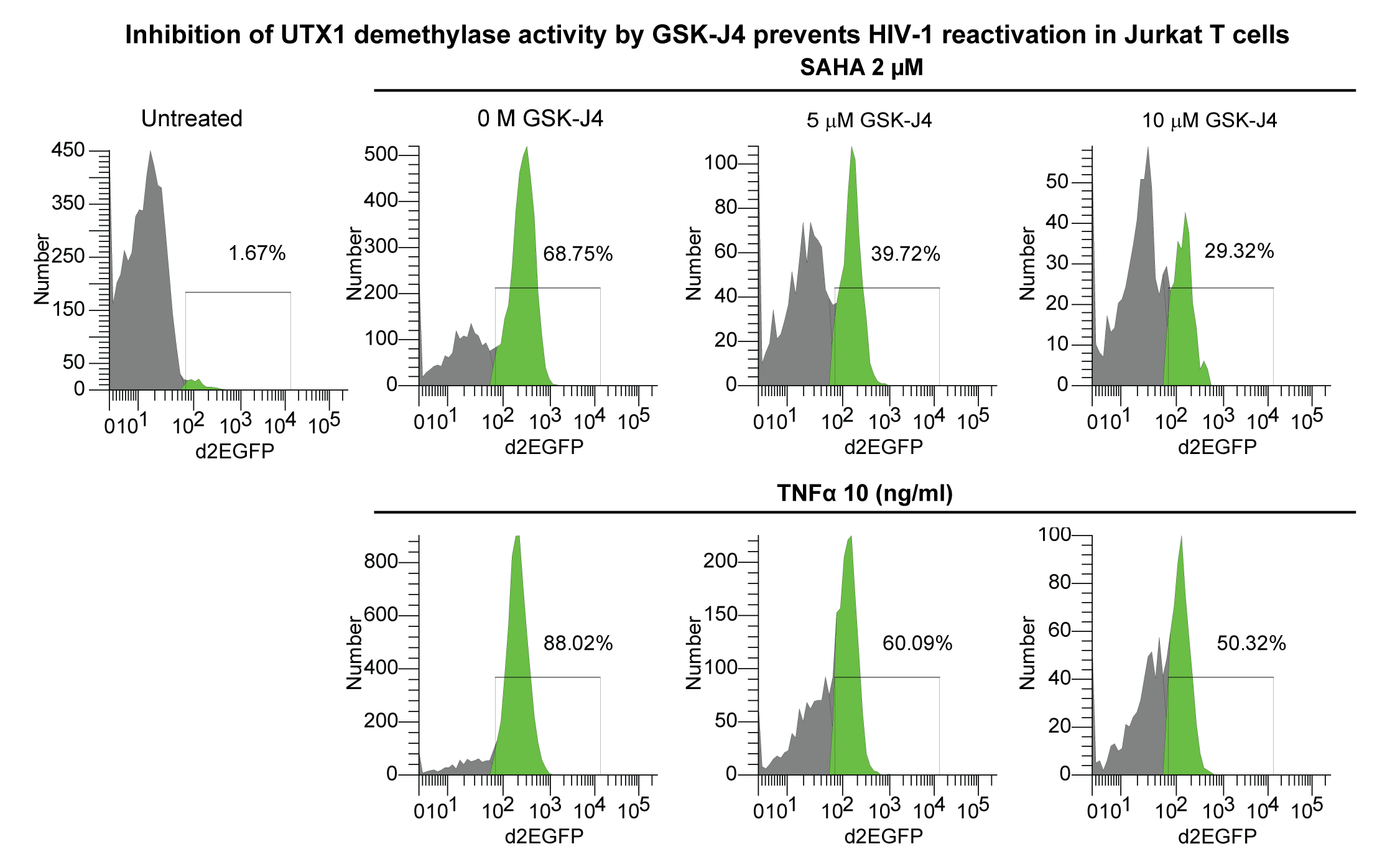

Supplement: S3 Fig — Representative FACS measuring d2EGFP expression in E4 cells are shown for cells pretreated with increasing concentrations of GSK-J4 for 24 hours and stimulated with SAHA (2 μM) or TNFα (10 ng/ml) overnight. (TIF) [file ppat.1010014.s003.tif]

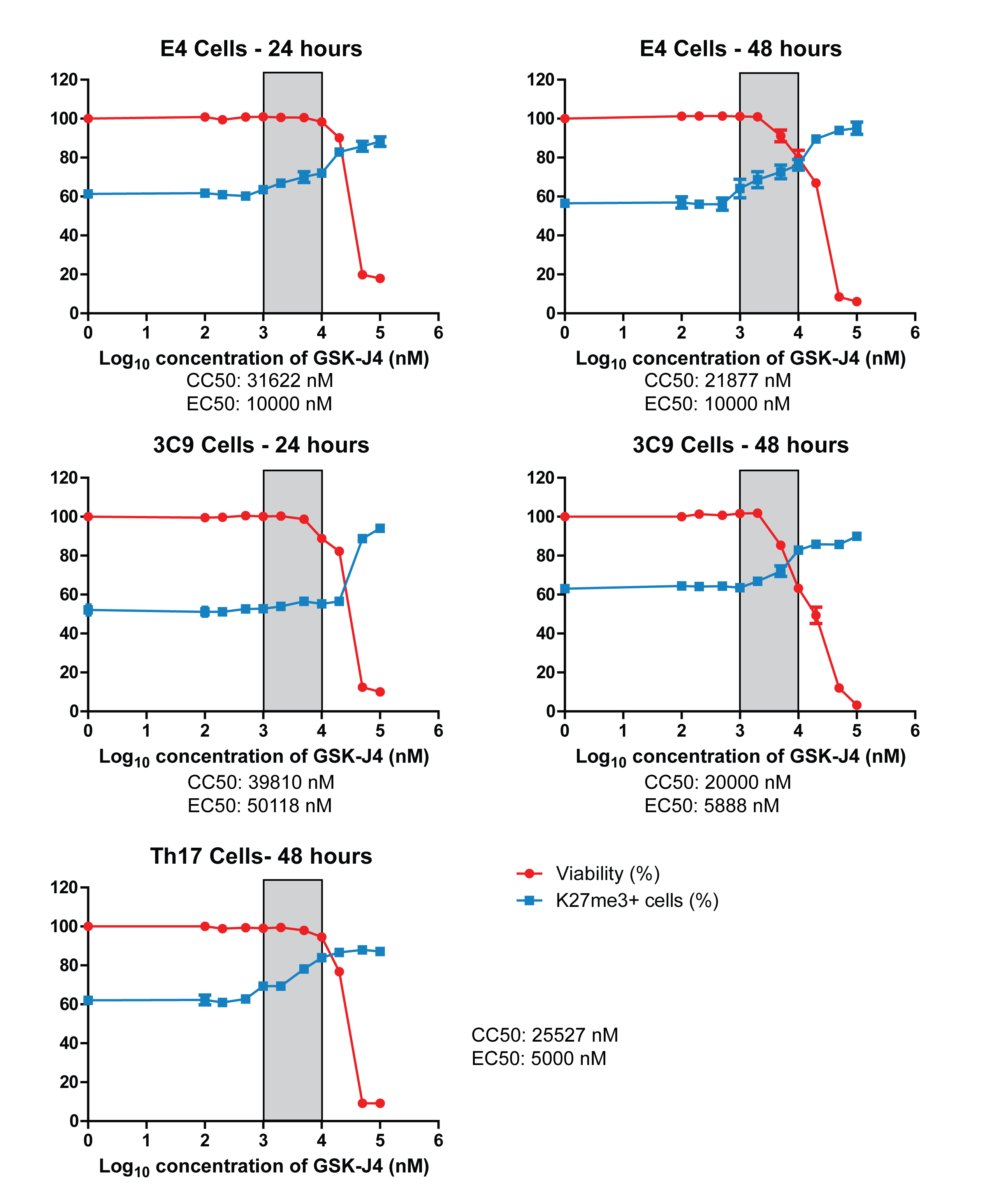

Supplement: S4 Fig — Cells were treated with increasing concentrations of GSK-J4 for 24 or 48 hours. Viability of cells was measured by FACs after being stained with Fixable Viability Dye eFluor 450 (#65-0863-14, ThermoFisher). Viability of cells was presented relative to cells treated with 0 nM of GSK-J4. Intracellular H3K27me3 levels of cells was measured by FACs using Tri-Methyl-Histone H3 (Lys27) antibody (#12158S, Cell Signalling) at a dilution of 1:500. (TIF) [file ppat.1010014.s004.tif]

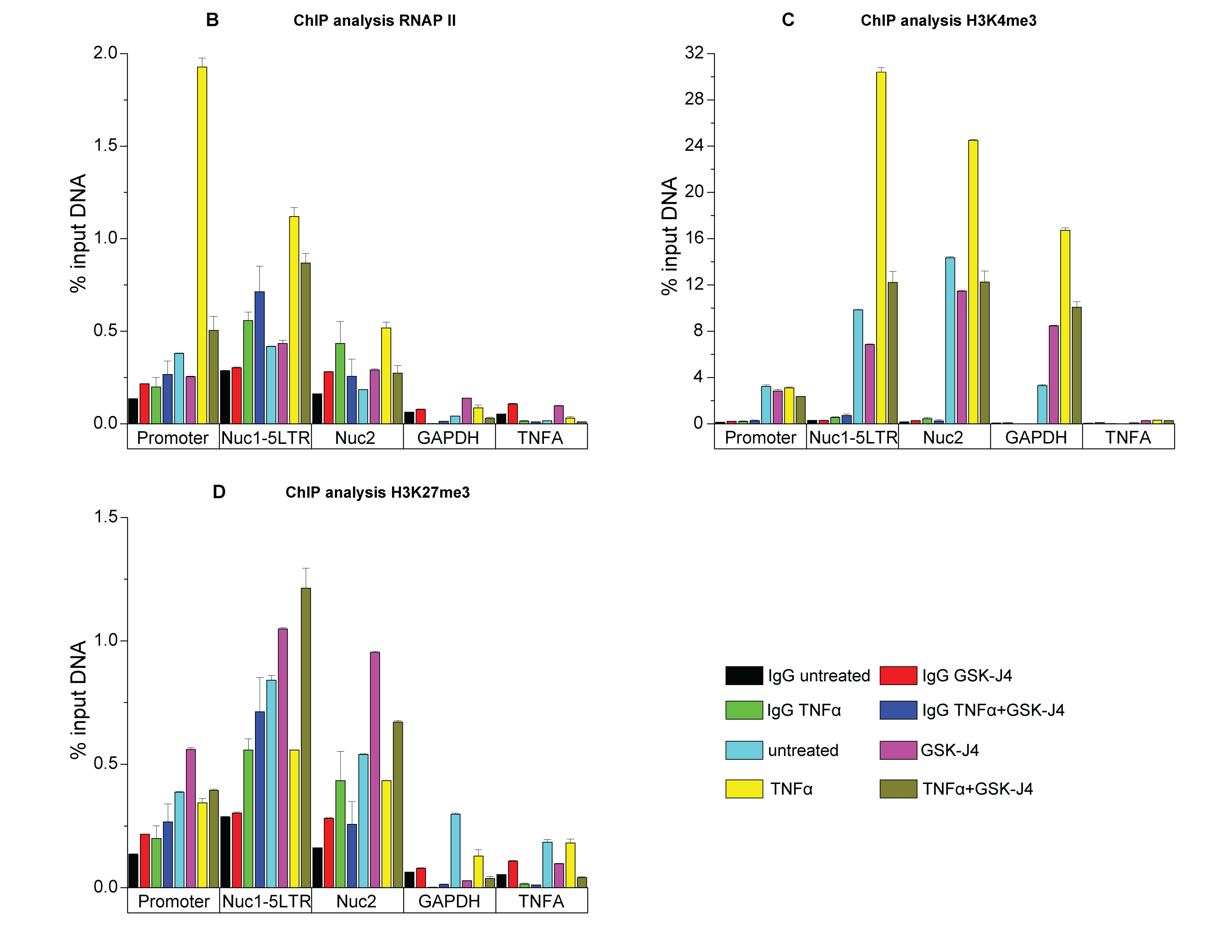

Supplement: S5 Fig — Enrichment of (B) RNAPII, (C) H3K4me3, and (D) H3K27me3 at the 5’LTR of HIV-1 when latent proviruses were left unstimulated or reactivated for an hour by TNFα (10 ng/ml) with or without the presence of GSK-J4 (5 μM). E4 cells were pretreated for 24 hours with GSK-J4 (5 μM) then further stimulated with or without TNFα (10 ng/ml) for an hour. Error bars: SEM of 3 separate quantitative real-time PCRs. (TIF) [file ppat.1010014.s005.tif]

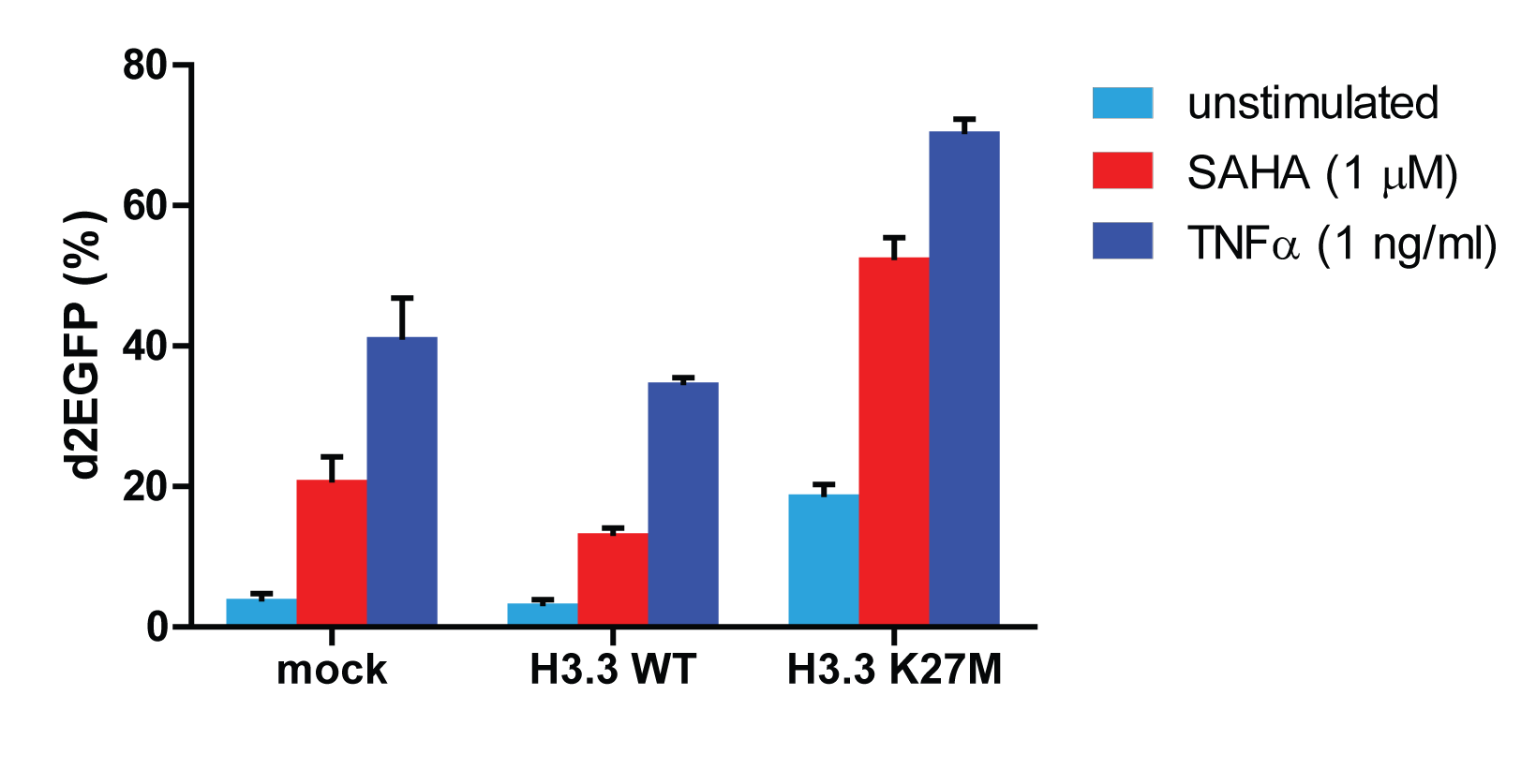

Supplement: S6 Fig — Cells were treated with SAHA (1 μM) or TNFα (1 ng/ml) overnight. HIV-1 reactivation was measured by FACs. Note that incorporation of WT H3.3 into HIV-1 chromatin does not predispose latently infected cells to viral reactivation. (TIF) [file ppat.1010014.s006.tif]

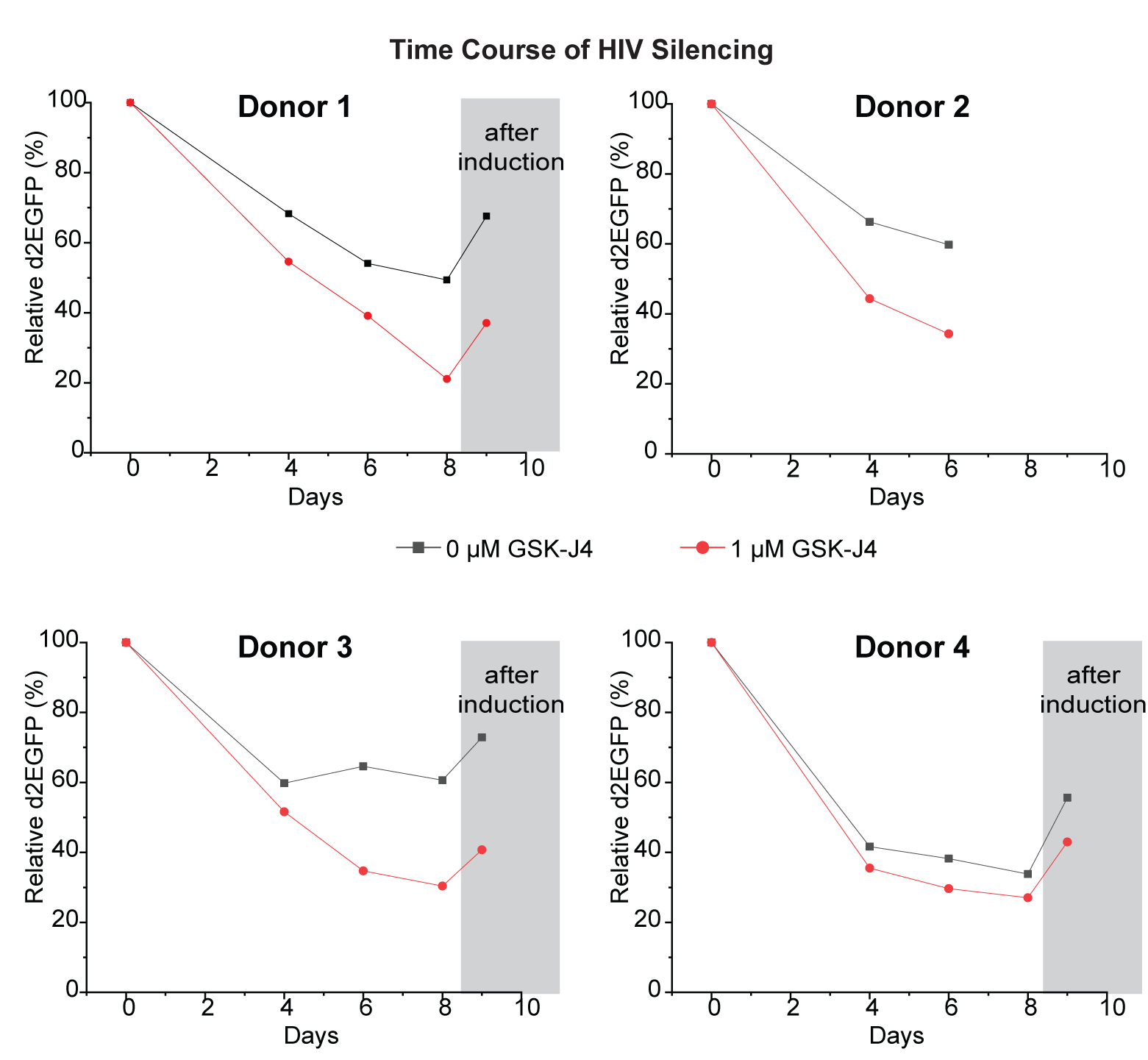

Supplement: S7 Fig — Data presented at each time point were the averages of duplicated measurements with FACs. Note that at Day 8, we did not have enough cells for collection with donor 2. (TIF) [file ppat.1010014.s007.tif]

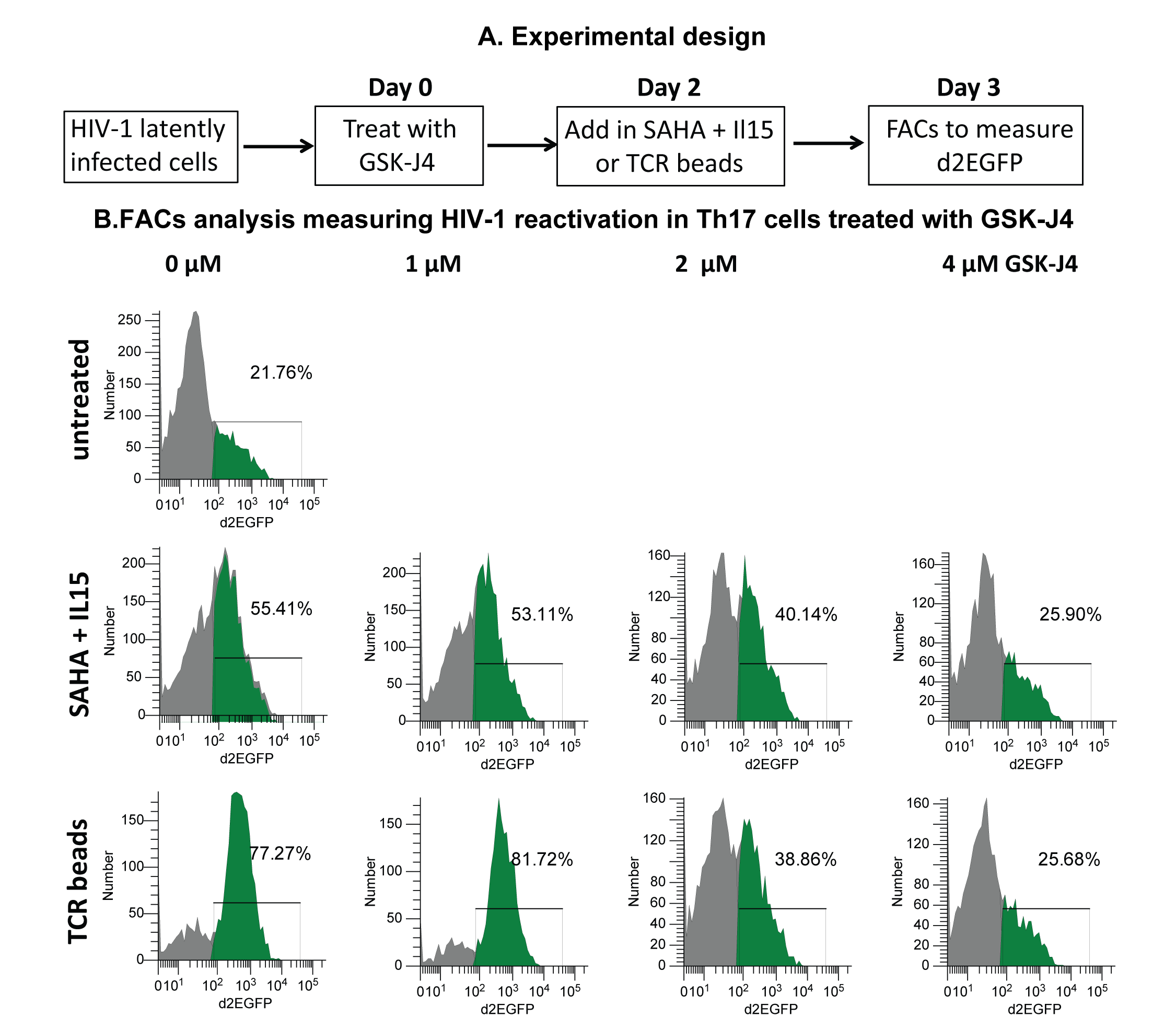

Supplement: S8 Fig — (A) Experimental design. (B) Flow cytometry measuring the reactivation of HIV-1 in Th17 cells by the combination of SAHA (1 μM) and IL15 (10 ng/ml) or Human T-Activator CD3/CD28 Dynabeads in the presence of increasing concentrations of GSK-J4. Cells were pretreated for 48 hours with GSK-J4, and then further stimulated overnight with SAHA & IL15 or Human T-Activator CD3/CD28 Dynabeads in a ratio of 25 μl of beads per 1 million cells. (TIF) [file ppat.1010014.s008.tif]

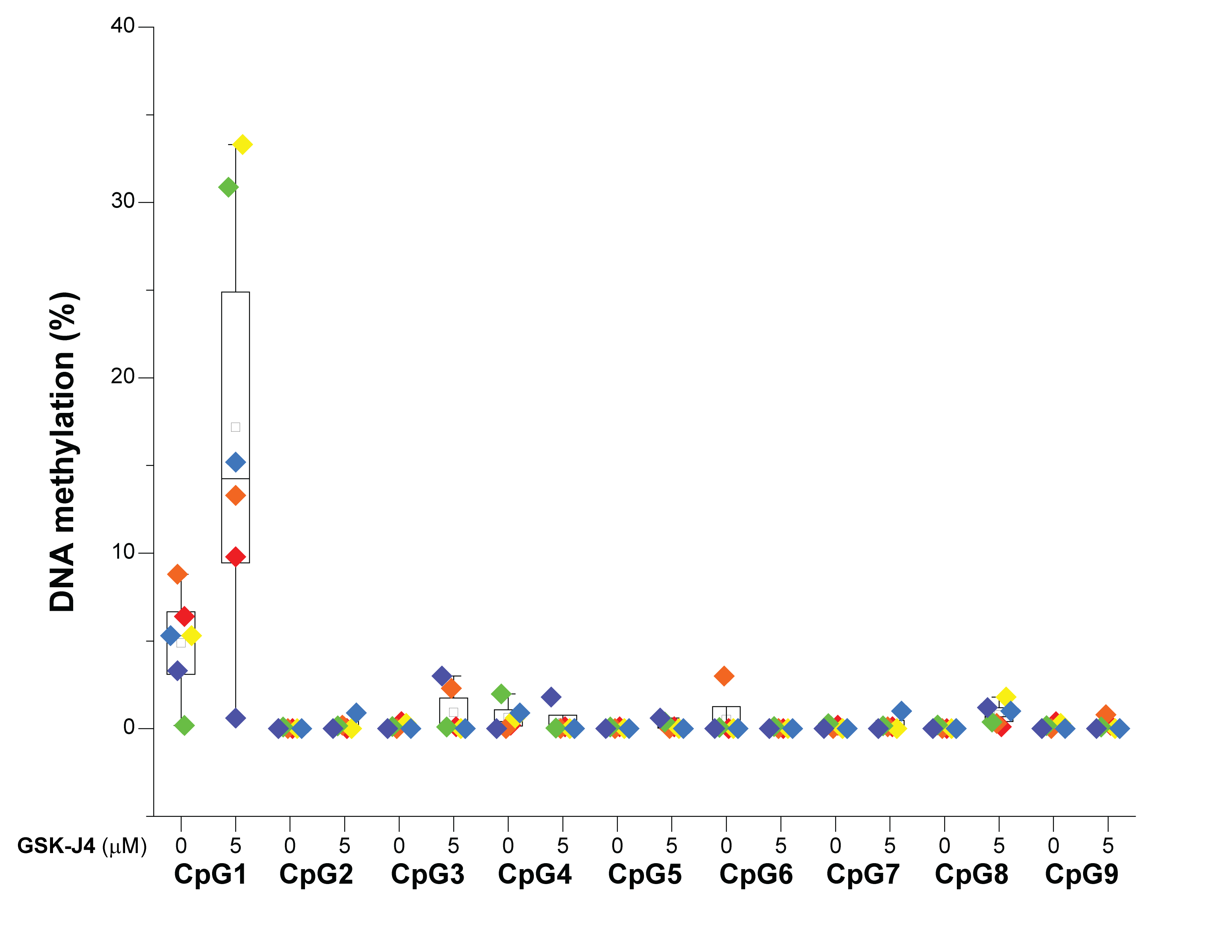

Supplement: S9 Fig — Note that these donors are the same as those presented in Fig 8B. (TIF) [file ppat.1010014.s009.tif]

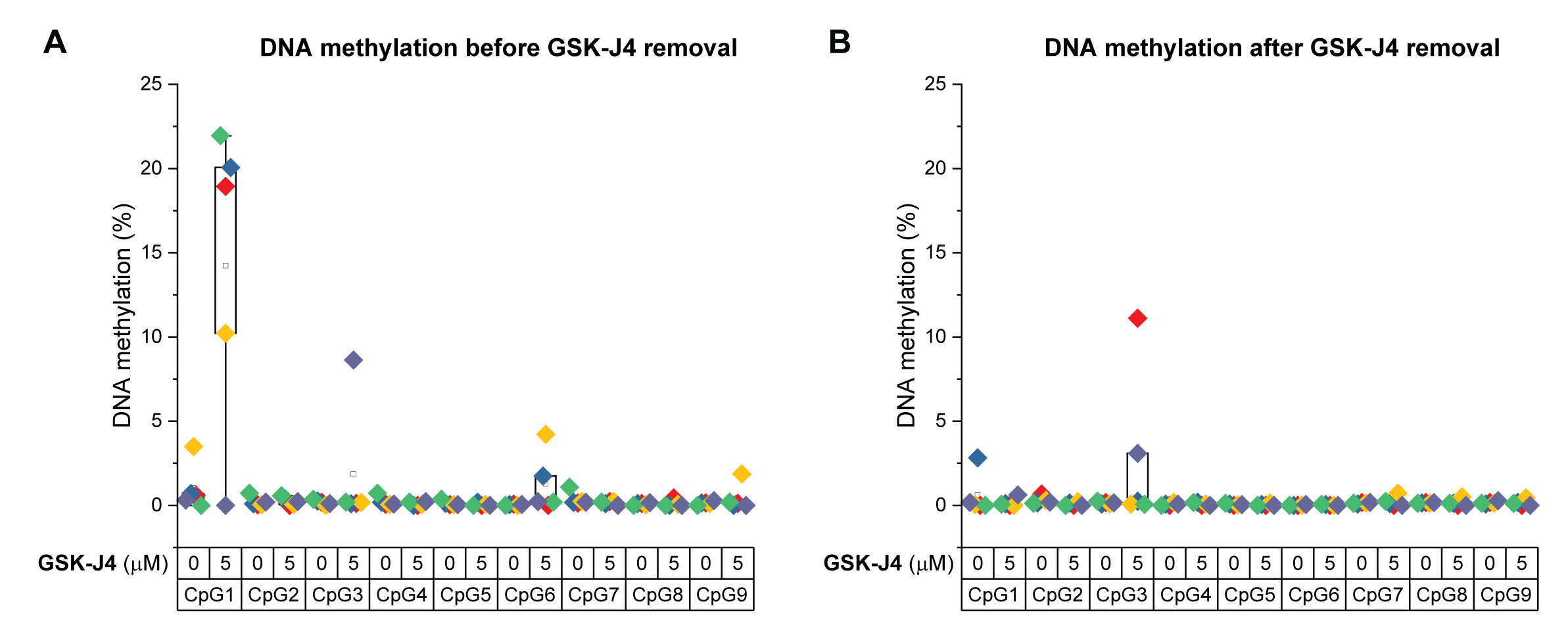

Supplement: S10 Fig — HIV-1 DNA methylation levels at individual CpG islands along the 5’LTR of HIV-1 before (A) and after (B) removal of GSK-J4. Experiments were performed on one HIV-1 donor with duplicates. (TIF) [file ppat.1010014.s010.tif]

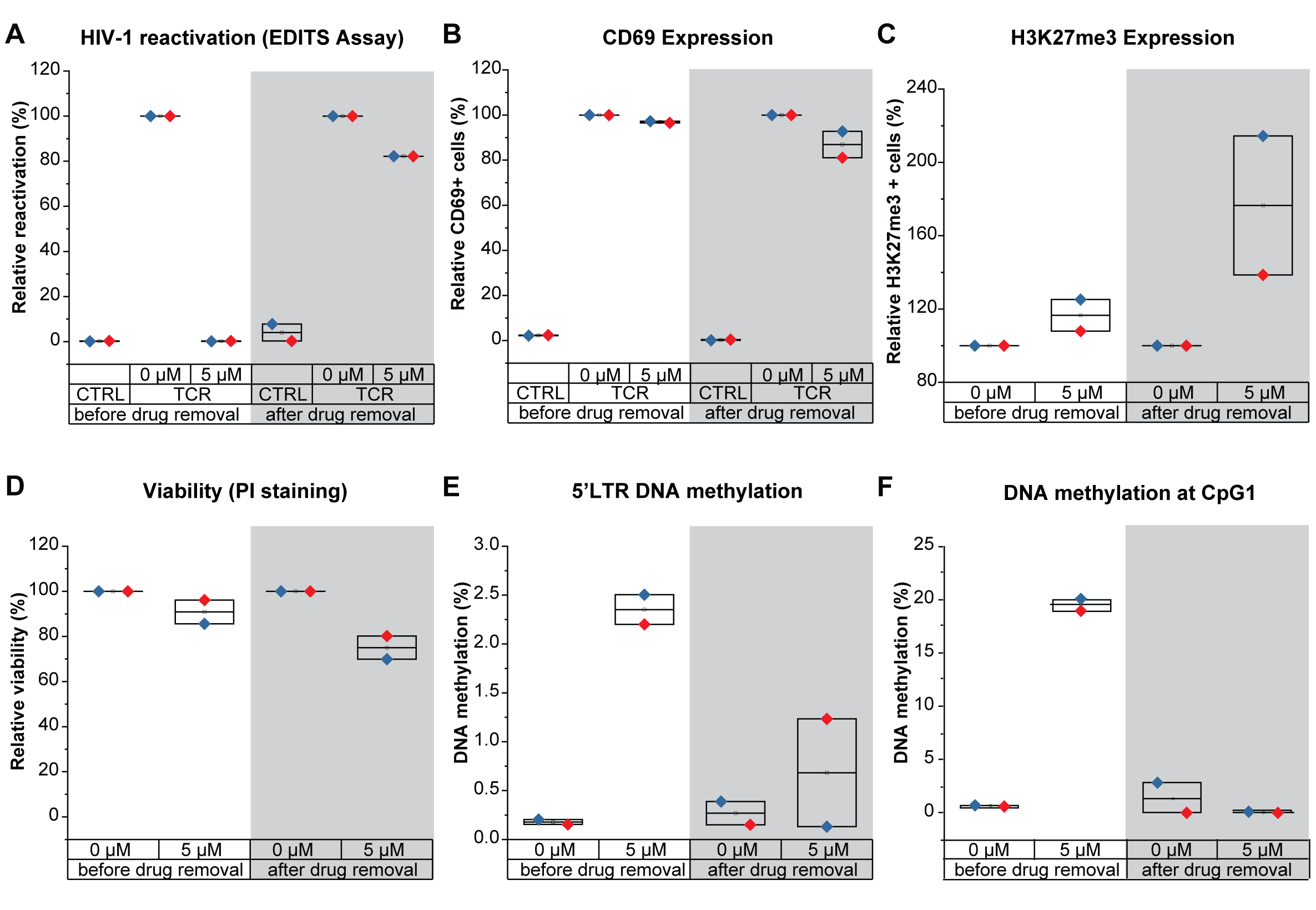

Supplement: S11 Fig — The impact of GSK-J4 on HIV-1 reactivation and DNA methylation was studied using the same experimental design as in Figs 7 and 8. Data shown is an analysis of two technical replicates from a single donor. (A) EDITs assay measuring HIV-1 reactivation in cells untreated or treated with GSK-J4 (5 μM) before and after GSK-J4 removal. Relative levels were normalized to the levels of spliced env mRNA from cells treated with T-Activator CD3/CD28 Dynabeads (presented as 100%) for each time point. Due to the limit of HIV-1 donor sample, experiments were performed on only one HIV-1 donor with duplicates. (B) Relative expression levels of CD69 from treated CD4+ memory T cells measured by FACS using CD69 antibody. Relative levels were normalized to the levels of CD69 from cells treated with T-Activator CD3/CD28 Dynabeads (presented as 100%) for each time point. (C) Relative intracellular H3K27me3 levels of treated CD4+ memory T cells measured by FACS using a H3K27me3 antibody. Cells were stained with Alexa Fluor 647-conjugated trimethyl histone H3 (Lys27) antibody (12158, Cell Signaling) and analyzed by FACS as described previously [22]. (D) Viability of cells by PI staining. After drug treatment, cells were stained with PI at the final concentration of 5 μg/ml for 5 minutes, then analyzed by FACS. (E) The average DNA methylation (as %) of CpGs at the 5’LTR CpG cluster from CD4+ memory T cells treated with the indicated concentration of GSK-J4 before and after GSK-J4 removal. (F) The percentage DNA methylation at the CpG island 1 at the 5’LTR CpG cluster of HIV-1 from CD4+ memory T cells induced with GSK-J4 before or after GSK-J4 removal. (TIF) [file ppat.1010014.s011.tif]
