# Supplementary material for: Inhibition of the H3K27 demethylase UTX enhances the epigenetic silencing of HIV proviruses and induces HIV-1 DNA hypermethylation but fails to permanently block HIV reactivation
Source: PLoS Pathog. 2021 Oct 21;17(10):e1010014. doi: 10.1371/journal.ppat.1010014 (PMC8562785; doi:10.1371/journal.ppat.1010014)
Supplement: Attachment — Submitted filename: KN_Reply to referees 28-Sep-2021.docx [file ppat.1010014.s013.docx]

Reviewer #1: Authors report on the inhibition of the H3K27me3 demethylase in an effort to epigenetically silence HIV transcription/reactivation. Although this block and lock approach seems to fail due to a transient effect, the elegant experiments increase the mechanistic understanding of both histone and DNA methylation at the transcription start site.

Reviewer #2: In this manuscript, the authors investigated the effects of UTX inhibition, an H3K27 demethylase, on HIV-1 latency and reversal from latency. They performed either shRNA experiments or a pharmacological approach (using a compound called GSK-J4) in several cellular models of HIV-1 infection or latency (T cell lines, a primary cell model of HIV-1 latency and patients cells isolated from HIV-1+ individuals). They showed that UTX inhibition induced HIV-1 latency by increasing the histone post-translational modification H3K27me3 while decreasing H3K4me3. Overall, the manuscript is well written. Several figures could be improved to provide a clearer message. In addition, despite the high interest to further understand the molecular mechanisms underlying HIV-1 latency, several additional experiments and justifications are needed to draw the conclusions stated by the authors.

Major comments:

- Figures 1B and 1C: the authors should present their results as a fold inductions compared to the result obtained in untreated condition.

**Answer:**

Generally, flow cytometric fluorescent data are presented in two ways:

1. Change in the percentage of positive or negative subpopulation of cells (**S1A** **Fig).**
2. Fold change in the mean fluorescence intensity (MFI) of the whole population of cells (**S1B** **Fig).** MFI is the arithmetic mean of fluorescence intensity of cells in the whole population. Data presented this way is similar to data reported from the luciferase or western blot assay.

When working with HIV, Tat induction results in high and general uniform expression levels leading to a well separated population of activated cells. Different levels of activators therefore primarily result in changes in the fraction of cells that express HIV markers (including GFP). This is in contrast to the types of results that are obtained using GFP expressed from a cellular promoter where the intensity of gene expression may shift over a large dynamic range. Therefore, for many years we have expressed HIV reactivation data in terms of the % positive cells.

To demonstrate that the results are similar with both methods of analysis, we plotted our data from the HIV-1 reactivation experiment performed on UTX knocked down E4 clones (**Fig. 1B**) in both manners in **S1 Fig.** Regardless of the way the data are reported, we observed the same inhibitory effect of UTX knock down on HIV-1 reactivation. Therefore, we chose to present HIV-1 reactivation data by change (or difference) in the percentage of subpopulation of cells positive for d2EGFP expression throughout the rest of the manuscript.

Indeed, the fold induction following SAHA treatment (Fig. 1C), compared to the untreated condition, is probably much higher in shUTX(1) and shUTX(2) cell lines than in the scrambled one. Regarding this result, the following conclusion made by the authors: “UTX shRNA sequences also prevented proviruses from being reactivated by SAHA stimulation” should be modified since high levels of viral reactivation occurred in shUTX(1) and shUTX(2) cell lines following SAHA treatment.

**Answer:**

We think the referee may have misunderstood the design of the experiment in Figure 1C. This data presents a series of individual clones infected with lentiviral vectors expressing control and UTX-1 shRNAs, so looking a fold expression is not really appropriate. Instead, the data shows that in a large subset of the UTX-1 shRNA treated cells basal proviral expression is severely restricted. Furthermore, the mean maximal fraction of activated cells is several reduced after treatment by SAHA. We have modified the text to clarify how this data was generated and clarify our conclusions.

- Figure 1B: the authors should present their data similarly in the 3 panels. In addition, it is unclear why CD3/CD28 stimulation has not been performed in 2D10 and 3C9 cells. Moreover, the lack of statistical analysis and the important error bars make the effect of UTX depletion on HIV-1 reactivation difficult to analyze (especially in the 2D10 cell line following SAHA treatment and in the 3C9 cell line following TNF-α stimulation).

Answer:

We modified **Fig. 1B** to present the data from each cell line in a similar format. To do so we removed the CD3/CD28 stimulation condition from the E4 cell panel. Most Jurkat cell lines respond very poorly to CD3/CD28 since they lose the T-cell receptor in culture. E4 cells were cloned and selective for CD3/CD28 responses, but even then, they are not as responsive as primary T-cells, perhaps because they also lack PTEN and other downstream regulatory factors. Statistical analysis has been added to the figure and is also given in **S1 Fig.**

- Figures 2 A-D: No negative control (such an IgG antibody) was used to assess background measurement in ChIP experiments (as well as for the other ChIP assays performed in the manuscript).

Answer:

The ChIP assays were performed using the Pierce Agarose ChIP Kit (Thermo Scientific) as mentioned in the Material and Methods. An IgG control antibody is included in the kit which we routinely included in our ChIP assays. The signals generated by IgG antibody were subtracted from the signals obtained with specific antibodies. To clarify this, we modified the Material and Methods section to state this explicitly. To allow interested readers to assess the data more fully the means and standard deviations of the ChIP signals from the IgG antibody compared to the specific antibodies have now been presented in **S2 Fig** and **S5 Fig**.

In addition, since Jurkat E4 cells have 2 LTRs, is it difficult to understand how the qPCR experiments using primers amplifying Nuc-0, Pro and Nuc-1 are specific of the 5’LTR without amplifying the 3’LTR.

Answer:

This is a generic problem when working with HIV. We agree with the reviewer that both the Nuc-0 and Pro primers we are using amplify both 5’LTR and 3’LTR and this unavoidable. However, the LTR is very polarized for transcription, so it is a safe assumption that the majority of the signal arises from the 5’ LTR and we detect GFP expression under our experimental conditions. We demonstrated asymmetric accumulation of RNAPII and other transcription elongation factors experimentally in an earlier study (Jadlowsky et al. Mol Cell Biol. 2014 Jun;34(11):1911-28. doi: 10.1128/MCB.01013-13. Epub 2014 Mar 17.), where we used LTR specific primers. Unfortunately, this requires the amplification of quite large regions that prevents distinction between the Nuc-0 and Pro regions. As a compromise, which also provides evidence for the favoring of the 5’LTR, we repeated the ChIP assays using a new set of Nuc-1 primers in which the reverse primer primes at a location specific to 5’LTR (the sequence of Nuc-1 reverse primer is also included in the Material and Methods). We have revised the text to discuss these issues.

- Figures 2 E-F: the authors explained that the shUTX(2) was less efficient than the shUTX(1). However, the experiments were performed using the shUTX(2) cell line while the shUTX(1) cell line was used in Figure 5. To strengthen their results, the authors should repeat their experiments using both shUTX cell lines or, at least, use the same shUTX cell line through the whole manuscript.

Answer:

This was an error. The shUTX(1) cell line was actually used in the experiments in Figure 2 and we have corrected the labeling in the **Fig 2**. The authors thank the reviewer for pointing this out.

- Figure 2B versus Figure 2F: the recruitment of UTX to the promoter region was much higher in the shScr cell line than in Jurkat E4 cells (0.1% compared to 22%). However, no explanation is provided regarding this variation. The authors should also performed ChIP assays in the shScr and shUTX(2) cell lines to assess the presence of H3K27me3 and correlate UTX depletion with H3K27me3 enrichment.

Answer:

The ChIP assays in **Fig 2B** were performed using Jurkat E4 cells, which are parental cells used to clone out the single clones transduced with scramble shRNA and UTX shRNA which were used in **Fig 2F.**

The ChIP assays in **Fig 2F** were performed on single clones of E4 cells transduced with scramble or UTX shRNAs. The variation in the recruitment of UTX in these two figures could be explained either on the basis of clonal selection or because the two experiments were performed at different times. This is also verified by different values of IgG controls noted in both experiments.

To confirm our results, we repeated ChIP assay in **Fig 2B** using the dual crosslinking method and presented this new data in Figure 2A. A detailed protocol has been included in the Material and methods.

- Figure 3A: GSK-J4 (100 microM) seems to increase viral transcription in E4 and 3C9 cell lines which is in contradiction with its putative repressive effect. However, this result is not explained by the authors. A similar observation is stated by the authors (lanes 228 - 229 in shUTX cells) without any explanation.

Answer:

There was an error in the Figure which we have now corrected. We have never used a concentration of 100 microM of GSK-J4 in any of our experiments. The authors apologize to the reviewer for any confusion generated by our error. To simplify the Figure, we have also replotted the data in two panels separating the E4 and 3C9 cells, which allows a straightforward assessment of the GSK-J4 block to HIV reactivation.

The observation mentioned in lines 228-229 was referring to Figure 5B. We clarified this and we further quote as follows: “Compared to the scrambled shRNA-treated control cells, we observed greater elevations of HIV-1 expression in the shUTX knock down cells, after 5-AZC treatment (**Fig 5B**). When treated with 5-AZC, cells expressing scrambled shRNA showed a less than a 1.5-fold increase in HIV-1 expression while the UTX shRNA treated cells resulted showed a 3 to 6-fold increase.” The authors apologize to the reviewer for any confusion.

- Figures 3B - D: The condition GSK-J4 alone should be included to evaluate the effects of this compound alone on HIV-1 latency (and not only in combination with TNF alpha) to assess if it is possible to reinforce viral latency.

Answer:

The experiment has been repeated with the requested condition as shown in **Fig 3B**. We observed a modest increase of H3K27me3 and decrease of H3K4me3 at HIV-1 LTR in response to the drug alone. However, this type of experiment is not designed to test whether GSK-J4 reinforces viral latency because we started with a population of cells where more than 95% harbored latent HIV-1 proviruses. We have addressed this issue later in the paper in our studies of the Th17 cells which is better model system to assess viral entry into latency.

In another experiment, we did observe higher levels of DNA methylation of HIV-1 upon treatment of cells with GSK-J4 (**Fig 5C**) and this is likely to reinforce viral latency.

- It is unclear why the authors decided to use a specific histone variant (H3.3) to introduce mutations instead of the “canonical” histone H3. Indeed, this histone variant is known to display a different chromatin environment often associated with euchromatin (reviewed by Sara Martire and Laura A. Banaszynski, Nature Reviews Molecular Cell Biology, 2020). Thus, the incorporation of the histone variant H3.3 along the HIV-1 provirus could make latently-infected cells more prone to viral reactivation.

Answer

We chose H3.3 because many published studies, including the reference the reviewer cited, have shown that H3.3 is associated with PRC2-dependent H3K27me3 deposition and gene expression regulation [1-3]. As reported in our previous papers, HIV-1 is Polycomb repressive complex 2 dependent [4] and it is therefore not surprising that H3.3 can be incorporated into HIV-1 chromatin. The fact that our experiments showed different degrees of HIV-1 reactivation in cells expressing different mutations of H3.3 proteins strongly supports this conclusion.

Furthermore, we did not observe any enhancement of viral reactivation in cells overexpressing wild type H3.3 versus mock control cells. The data have been included in **S6 Fig**.

In this specific experiment, we did not base our conclusion on the comparison of HIV-1 reactivation in cells overexpressing wildtype H3.3 variant versus mock cell lines (cells transduced with empty vector). Instead, we compared the levels of HIV-1 reactivation in cells expressing different H3.3 mutants: wild type, K4M, K27M, and K4M-K27M. Therefore, our experiment is appropriately controlled and our conclusions are still valid even if the incorporation of the histone variant H3.3 along the HIV-1 provirus could make latently-infected cells more prone to viral reactivation.

- Figure 5D: Western blot assay shows that the level of DNMT3A was very low in the T-cell line used by the authors. It is therefore less physiologically relevant to assess the role of DNMT3A as a putative DNA methylation effector of the HIV-1 5’LTR. The authors should also evaluate the role of DNMT3A in cell lines expressing higher level of DNMT3A.

Answer:

The Western blots shown in the figure were exposed for a brief period to avoid overexposing the overexpressed DNMT3A in the transfected cells. This may have created the erroneous impression that the cell line we were using expressed low levels of intrinsic DNMT3A. We subsequently repeated the western blots with longer exposures to show the levels of DNMT3A in the host cells and used these blots for the revised Figure 5.

We chose to overexpress Flag-tagged DNMT3A because we could not find a good ChIP-graded antibody against DNMT3A. Flag-tagging DNMT3A allowed us to use anti-Flag antibody, which with its improved immunoprecipitation, allowed for better ChIP signals. In addition, our observation is also only based on the comparison of signals between cells overexpressing DNMT3A which are untreated (light blue bar) or treated with GSK-J4 (dark blue bar).

- Figure 6D: the authors performed meDIP assay at day 4 post-treatment while the highest HIV-1 silencing effect was obtained at day 8 post-treatment. Why the authors did not evaluate levels of DNA methylation at day 8 post-treatment should be clarified.

Answer:

The experiments were performed using the QUECEL primary Th17 cell model of HIV latency [4]. When working with primary cells the number of HIV-1 cells that can be collected are limited and we have to make some trade-offs in the experimental design. Due to the limited number of cells from healthy donors, we had to retain enough cells for Western blot experiments in **Fig 6B**, FACS analysis of GFP in duplicates at each of the time points shown, and DNA isolation for MeDIP experiments at day 4 post-treatment for each individual donor. Furthermore, the numbers of cells isolated from each individual donor varied.

Therefore, while we agree with the reviewer that for an ideal scenario, the MeDIP experiment should have been performed at day 8 post-treatment by day 8 post-treatment, we had insufficient cells for the MeDIP experiment (which require at least 2 million cells per IP). This also accounts for our lack of data at day 8 post-treatment for one of the donors in the experiment.

Minor comments:

- Figures 3B - D: the authors should explain why they used GAPDH and TNFA as controls.

Answer

GAPDH and TNFA were chosen to show that changes of H3K4me3 and H3K27me3 levels are HIV-1 specific and not detected at GAPDH and TNFA, which are activated by TNF-α but are not documented as bivalent genes. This has now been stated in the text.

- Figure 4B: the authors should explain why statistical analyses were performed compared to the H3.3 K27M condition and not to the H3.3 wild-type condition.

Answer

We chose to present the statistical analyses this way to emphasize the necessity of H3K27me3 depletion on HIV-1 reactivation and the indispensable nature of H3K4me3 on HIV-1 reactivation mediated by H3K27me3 depletion. Detailed statistical analyses with different pairs of treatments are now included in supplemental **Table 1**.

- Figure 5B: since all the cells were treated with 5-aza, the reviewer assumes that the legend (grey and purple) corresponds to shScr and ShUTX(1), respectively. The authors should confirm this.

Answer

We confirmed that the legend corresponds to shScr and shUTX(1). We thank the reviewer for helping us correct this mistake.

- Cellular viability following GSK-J4 treatment should be assessed in cell lines and not only when using patient cell cultures.

Answer

Cellular viability following GSK-J4 treatments on different cells is now shown in **S4 Fig**. As stated in the Material and methods section, HIV-1 reactivation in cells was quantified with FACS by gating only on viable cells.

Reviewer #1: Major questions

1. My major question relates to the pharmacology of the GSK-J4 inhibitor. The compound is used at different concentrations in different cells and cell lines and only once its effect on the viability of the cells is measured (Figure7). Authors should present data on IC50 and CC50 values and dose response curves in the different cells and cell lines used.

Answer:

Data on IC_50_ and CC_50_ values of GSK-J4 on E4, 3C9 cells and Th17 primary T cells have been presented in **S4 Fig**. Although the drug shows high toxicity at high concentrations, we are working in an experimental range where toxicity is acceptable. Ironically, the drug is more effective and toxicity is reduced in the primary cells, perhaps because these cells replicate more slowly.

2. Authors downplay their own approach because of its transient effect, and imply that the effect of didehydrocortistatin A is not due to epigenetic silencing. This is an indirect conclusion at best since no data are shown with the latter compound. In fact very few data (only on DNA methylation) are given to demonstrate the transient effect of GSK-J4 (only experiment 8?). This ought to be better elaborated. Can authors exclude that more permanent changes in the histone epigenetic environment may exist? Line 357 In a similar way, they could have tested if withdrawal of GSK-J4 reactivated HIV.

Answer:

We have performed additional experiments to compare levels of HIV-1 reactivation and DNA methylation at HIV-1 5’LTR before and after GSK-J4 withdrawal. Due to the high numbers of cells required for these experiments and the limited number of HIV-1 donor samples with sufficient numbers of cells that we currently have available, we were only able to perform those experiments on one HIV-1 donor with duplicates. The new data are presented in **S11** **Fig**. Before GSK-J4 withdrawal, levels of HIV-1 reactivation measured by EDITs assay were completely inhibited with GSK-J4 treatment. After GSK-J4 withdrawal, levels of HIV-1 reactivation in cells treated previously with GSK-J4 were restored up to 80% compared to those from cells untreated **(S11 Fig A)**. Levels of CD69 did not vary much between untreated cells and cells treated with GSK-J4 before or after GSK-J4 withdrawal **(S11 Fig B)**. Somewhat surprisingly, we see a somewhat persistent increase in the global levels of H3K27me3 in cells treated with GSK-J4 after GSK-J4 withdrawal, indicating that turnover rate of H3K27me3 was more than 72 hours **(S11 Fig C)**. The viability of cells treated with GSK-J4 was slightly decreased after drug withdrawal **(S11 Fig D)**. Levels of HIV-1 DNA methylation at 5’LTR or CpG1 were highly increased in cells treated with GSK-J4 before drug withdrawal; however, after drug withdrawal, DNA methylation of HIV-1 in cells treated with GSK-4 was restored to levels similar to untreated cells at both at the 5’LTR and CpG1 **(Fig 8B & C, S11 Fig E & F)**. Taken together, the data confirmed the transient effect of GSK-J4 on the inhibition of HIV-1 reactivation and induction of HIV-1 DNA methylation; even though its effect on inhibition of UTX activity might still be maintained globally 72 hours after drug removal. However, H3K27me3 at HIV-1 has been known not to resist to reactivation by strong stimulus like TCR. In addition, this is a global measurement of H3K27me3 in cells but not a measurement of H3K27me3 at HIV-1 chromatin. While we are not disputing the data on dCA presented by the Valente and colleagues, we are skeptical about their conclusion that the inhibitory effect of dCA on HIV-1 reactivation is due to epigenetic silencing.

Reviewer #1: Minor remarks

1. Figure 7, show specific data on other CpG islands in Suppl Figures (now only CpG-1 is shown)

Answer:

Those data have now been included in **S9 Fig.**

2. p21 line 420-424 If H3.3 K4M inhibits K27M reactivation why does H3.3K27me3 removal occur first?

Answer:

We agree that H3.3K27me3 removal does not necessarily occur first based on our experimental data. We have corrected this in the indicated discussion section.

3. Fig 1B Why is there a difference in the effect of UTX knockdown if Tat is inactive? Explain in the discussion.

Answer:

H13L is a functional Tat, which was originally identified in the sequence of latently infected cells. We can easily reactivate HIV-1 in 2D10 cells by many different stimuli [5, 6]. We clarified this in the text.

4. Fig 3A: the concentrations in the X-axis are far from clear

Answer:

There was an error in the construction of the graph. The data have been presented correctly in a new **Fig 3A**.

5. In line 57, the authors refer to block-and-lock as a strategy to eliminate the latent provirus but this strategy does not eliminate the latent provirus.

Answer:

The authors agree with the reviewer that block-and-lock does not eliminate the latent provirus. The text has been corrected as follows: “There are two major strategies for eliminating latent proviruses or the rebound of latent proviruses.”

6. Line 142. Did authors also look at accumulation of H3K4 methylation at promotor in UTX KD cells since UTX also influences H3K4me3 levels? In next section they report these results with the UTX inhibitor but did they see this also in UTX KD cell lines?

Answer:

This experiment has been performed and included in **Fig 2B**. We observed a modest decrease in H3K4me3 levels after UTX knock down, similarly to when we treated cells with GSK-J4.

7. Tet1/ Tet2 KD not so convincing effect.

Answer:

We repeated the experiment, and the new data have been presented in the new **Fig. 5**. We speculate that to acquire a more profound inhibitory effect, simultaneous knock out of Tet proteins will be required. Unfortunately, we have not successfully knocked out both TET1 and TET2 in a single cell, perhaps due to toxic effects. However, the observed inhibitory effects of knocking out individual TET proteins on HIV-1 reactivation suggested Tet proteins actively demethylate HIV-1 DNA, which prevents long-term methylation of HIV-1 DNA. We also elaborate this further as following.

8. Fig 6B and C. In each experiment 4 donors are tested, are these the same donors? In Fig 6B they show the individual donors; in 6C they average the donors. It may be interesting to see if the extent of H3K27me3 accumulation correlates with HIV silencing among the different donors. If they tested the same donors, they could show the individual donor colours as well in Fig6C.

Answer:

The same set of four donors was used for Fig 6B and D and they are now color coded to identify each donor. With this limited sample size, it is not possible to establish a correlation between H3K27me3 and HIV silencing.

The data in **Fig. 3** is the time course for shutdown as measured by GFP expression and this represents averaged data. We separated the individual donors as recommended by the reviewer in **S7 Fig.**

9. Line 357 In the introduction they mention that DNA methylation is a more permanent, long term effect. How does this correlate with the strong decline of methylated DNA after only 72h after cpd removal?

Answer:

Evidence that DNA methylation is a more permanent, long term effect is based on studies of DNA methylation performed on human endogenous retroviruses (HERVs) and imprinted genes. We speculated that if we could similarly induce enhanced DNA methylation of latent HIV-1, we would be able to suppress latency for the long term. We have clarified the statement in the introduction.

Very limited studies of DNA methylation have been done on latent HIV-1 from infected donors. Therefore, the strong decline of methylated DNA after only 72h after drug removal is quite striking. It implies that a different mechanism governs DNA methylation between HIV-1 and HERVs or imprinted genes. Two features of HIV-1 likely protects HIV-1 from long-term DNA methylation: the bivalency of its LTR and the active involvement of TET proteins in DNA demethylation of HIV-1. The bivalency of HIV-1 LTR is characterized by the presence of both H3K27me3, which is deposited by PRC2 group, and H3K4me3 marks. PRC2 recruitment and H3K4me3 presence have been shown to provide gene promoters protection from DNMT-mediated DNA methylation [7]. Reduction of H3K4me3 levels at HIV-1 LTR by UTX knock down or GSK-J4 treatment erases the protection from DNMTs and recruits DNMT3A to HIV-1, which in turn results in elevated DNA methylation of HIV-1. In addition, the active DNA demethylation of HIV-1 by TETs is another factor protecting latent HIV-1 LTR from DNA hypermethylation. We speculate that TET proteins might be more active on HIV-1 DNA demethylation than in HERVs or imprinted genes because of the architectural difference of their chromatin. Different TET proteins might redundantly govern HIV-1 DNA demethylation. This is reflected in the observation that knock out of individual TET1 or TET2 inhibits HIV-1 reactivation. Our observations are strongly relevant regarding the essential roles of TET proteins in development, proliferation, and differentiation of T cells, including CD4+ T cells [8-10]. We have included a discussion of these issues in the revised manuscript.

References

1.	Martire S, Banaszynski LA. The roles of histone variants in fine-tuning chromatin organization and function. Nat Rev Mol Cell Biol. 2020;21(9):522-41. Epub 2020/07/16. doi: 10.1038/s41580-020-0262-8. PubMed PMID: 32665685; PubMed Central PMCID: PMCPMC8245300.

2.	Banaszynski LA, Wen D, Dewell S, Whitcomb SJ, Lin M, Diaz N, et al. Hira-dependent histone H3.3 deposition facilitates PRC2 recruitment at developmental loci in ES cells. Cell. 2013;155(1):107-20. Epub 2013/10/01. doi: 10.1016/j.cell.2013.08.061. PubMed PMID: 24074864; PubMed Central PMCID: PMCPMC3838450.

3.	Wang Y, Long H, Yu J, Dong L, Wassef M, Zhuo B, et al. Histone variants H2A.Z and H3.3 coordinately regulate PRC2-dependent H3K27me3 deposition and gene expression regulation in mES cells. BMC Biol. 2018;16(1):107. Epub 2018/09/27. doi: 10.1186/s12915-018-0568-6. PubMed PMID: 30249243; PubMed Central PMCID: PMCPMC6151936.

4.	Nguyen K, Das B, Dobrowolski C, Karn J. Multiple Histone Lysine Methyltransferases Are Required for the Establishment and Maintenance of HIV-1 Latency. mBio. 2017;8(1). Epub 2017/03/02. doi: 10.1128/mBio.00133-17. PubMed PMID: 28246360; PubMed Central PMCID: PMCPMC5347344.

5.	Pearson R, Kim YK, Hokello J, Lassen K, Friedman J, Tyagi M, et al. Epigenetic silencing of human immunodeficiency virus (HIV) transcription by formation of restrictive chromatin structures at the viral long terminal repeat drives the progressive entry of HIV into latency. J Virol. 2008;82(24):12291-303. Epub 2008/10/03. doi: 10.1128/JVI.01383-08. PubMed PMID: 18829756; PubMed Central PMCID: PMCPMC2593349.

6.	Sharma AL, Hokello J, Sonti S, Zicari S, Sun L, Alqatawni A, et al. CBF-1 Promotes the Establishment and Maintenance of HIV Latency by Recruiting Polycomb Repressive Complexes, PRC1 and PRC2, at HIV LTR. Viruses. 2020;12(9). Epub 2020/09/24. doi: 10.3390/v12091040. PubMed PMID: 32961937; PubMed Central PMCID: PMCPMC7551090.

7.	Rasmussen KD, Helin K. Role of TET enzymes in DNA methylation, development, and cancer. Genes Dev. 2016;30(7):733-50. Epub 2016/04/03. doi: 10.1101/gad.276568.115. PubMed PMID: 27036965; PubMed Central PMCID: PMCPMC4826392.

8.	Tsagaratou A, Lio CJ, Yue X, Rao A. TET Methylcytosine Oxidases in T Cell and B Cell Development and Function. Front Immunol. 2017;8:220. Epub 2017/04/15. doi: 10.3389/fimmu.2017.00220. PubMed PMID: 28408905; PubMed Central PMCID: PMCPMC5374156.

9.	Nakatsukasa H, Oda M, Yin J, Chikuma S, Ito M, Koga-Iizuka M, et al. Loss of TET proteins in regulatory T cells promotes abnormal proliferation, Foxp3 destabilization and IL-17 expression. Int Immunol. 2019;31(5):335-47. Epub 2019/02/07. doi: 10.1093/intimm/dxz008. PubMed PMID: 30726915.

10.	Yue X, Lio CJ, Samaniego-Castruita D, Li X, Rao A. Loss of TET2 and TET3 in regulatory T cells unleashes effector function. Nat Commun. 2019;10(1):2011. Epub 2019/05/03. doi: 10.1038/s41467-019-09541-y. PubMed PMID: 31043609; PubMed Central PMCID: PMCPMC6494907.
